# Micro- and Nanoplastics Act as Metal Carriers with the Potential to Alter Human Gene Expression Patterns—The Inferences from Bioinformatic Online Tools

**DOI:** 10.3390/biom15101418

**Published:** 2025-10-06

**Authors:** Maja Grabacka, Małgorzata Pierzchalska

**Affiliations:** Department of Biotechnology and General Technology of Foods, Faculty of Food Technology, University of Agriculture, ul. Balicka 122, 30-149 Cracow, Poland; malgorzata.pierzchalska@urk.edu.pl

**Keywords:** microplastics, nanoplastics, polymer particles, iron, copper, zinc, gene clusters, ferroptosis

## Abstract

Micro- and nanoplastic particles (MNPLs) present in the environment have recently become a potential health hazard factor due to the ability to penetrate living organisms, their organs, and cells. MNPLs interact with and absorb chemicals and elements, including metals, such as iron, copper, and zinc, and transport them into the cells. The cells subsequently respond with the altered gene expression profiles. In this study, we applied freely accessible online bioinformatic tools to draw out the sets of genes modulated by the metal ions and MNPLs. We focused on the gene interactome as revealed by The Comparative Toxicogenomics Database (CTD). To achieve a deeper insight into the biological processes that are potentially modulated, the retrieved CTD lists of genes, whose expression was influenced by MNPLs and metals, were subsequently analyzed using online tools: Metascape and String database. The genes from the revealed networks were arranged into functional clusters, annotated mainly as inflammation and immune system activity, regulation of apoptosis, oxidative stress response, Wingless-related Integration Site (WNT) signaling and ferroptosis. The complexity of the interactions between the gene sets altered by MNPLs and metal ions illustrates their pleiotropic effects on living systems.

## 1. Introduction

Nowadays, particulate plastic contamination of the environment is considered one of the emerging threats to human health, and some experts suggest that humanity is now on the verge of a “plastic crisis” (World Health Assembly (WHA) Resolution 76.17, 2023) [1]. Particulate plastics refer to plastic particles smaller than 5 mm in length or diameter, generally invisible to the naked human eye. Such pollutants are commonly divided into two classes: microplastics (bigger than 1 μm) and nanoplastics (smaller than 1 μm). We use the term ‘micro- and nanoplastics’ (MNPLs) throughout this publication to denote particulate plastics that can be absorbed by cells. MNPLs may come in multiple forms and originate from various sources. It can be secondary (products of large pieces, e.g., plastic bottles or bags, fragmentation and degradation) or primary (the effect of the direct production process of fabrics, cosmetics, domestic cleaning products, dyes, and others). Particulate plastics found in terrestrial and aquatic environments or the atmosphere could be in the form of fibers or spherical, hexagonal, square, triangular, and irregular fragments or beads [2]. Humans eat, drink, and inhale plastic particles every day, and it is estimated that an average person consumes between 78,000–211,000 such units per year [3].

The main idea behind our current study is to analyze the microplastic influence on mammalian cells in the context of possible disturbances in metal ion intracellular homeostasis. To achieve that aim, we have conducted a thorough literature and genetic database search, and pursued bioinformatic analysis with selected freely accessible online tools to project the networks of regulated genes.

## 2. Metal Ions as Micronutrients Important in the Regulation of Cell Physiology

Metal ions are crucial micronutrients that are necessary for the multitude of biochemical processes carried out in living cells. Nevertheless, free transition metal ions (particularly copper and iron) are extremely toxic and likely to catalyze the oxidoreductive reactions that produce reactive oxygen and nitrogen species, deleterious to virtually all cellular components. Metal ion homeostasis is tightly regulated at the level of plasma membrane systems for uptake or export, as well as intracellular storage proteins and chaperones. The dysregulation of these systems leads ultimately to cell death through entering numerous paths: apoptosis, ferroptosis, or recently discovered cuproptosis and lysozincrosis. These cell death types are frequently associated with the inability to cope with oxidative stress and the maintenance of redox balance.

Metal ions participate in multiple cellular processes, not only acting as secondary messengers (Ca^2+^), coenzymes (Cu^2+^, Zn^2+^, Mn^2+^, Mg^2+^) or indispensable elements of the prosthetic groups (heme iron, Fe-S clusters), but they also stabilize ternary structures of proteins. In the following sections, we briefly summarize the general roles of the most common ions in cell biology.

### 2.1. Calcium

In the endoplasmic reticulum and Golgi cisterns, concentrations of calcium ions oscillate between 200 and 650 µM, whereas in the cytoplasm, a low concentration (~100 nM) is maintained [4]. This difference enables rapid signaling through receptor tyrosine kinases or G-protein-coupled plasma membrane receptors [5]. Phospholipase C (PLC), when activated by upstream receptors, hydrolyses a membrane phospholipid phosphatidylinositol-4,5-bisphosphate into diacylglycerol (DAG) and inositol 1,4,5-triphosphate (IP3). IP3 interacts with calcium channels in the endoplasmic reticulum (ER). This interaction leads to the opening of the channels and allows flux of calcium into the cytoplasm, thereby activating multiple downstream targets [6].

Mitochondria also store calcium ions. The close vicinity of their membranes to the ER and its [Ca^2+^] channels facilitates the uptake of the released calcium through the mitochondrial calcium uniporter (MCU) [7]. Mitochondrial Ca^2+^ overload is a signal for the opening of the mitochondrial permeability transition pore (mPTP) channel, which leads to uncontrolled flux of various small molecules through the pore, collapse of mitochondrial membrane potential, swelling, and subsequent release of important functional components, and, ultimately, to apoptotic death [8].

### 2.2. Magnesium

Magnesium is one of the most abundant ions in living cells, reaching concentrations in the range of 15 and 20 mM [9,10], and is essential for the activity of over 350 enzymes [9]. The role of magnesium in catalysis comprises stabilization of the intermediate products, facilitation of the physical contact between the substrates, and increasing the proximity of the reactants and stabilizing the chemical groups detached from the substrate [11]. Furthermore, Mg^2+^ is necessary for stabilization of ATP, which is important for sustaining energy metabolism, including oxidative phosphorylation [12]. Interestingly, the intracellular calcium and magnesium management is interconnected: Mg^2+^ regulates uptake of Ca^2+^ ions via ER through Ca^2+^-ATP-ase and Ca^2+^ release through activity of IP3 receptors [13].

### 2.3. Iron

Iron is an essential trace element, which in the form of ferrous ions (Fe^2+^) builds heme prosthetic groups and Fe-S clusters, necessary for a large number of enzymes that participate in cellular respiration, the Krebs cycle, tRNA maturation, DNA replication and repair, and numerous other processes indispensable for living cells [14,15,16]. Nevertheless, the excess of intracellular iron is toxic and contributes to the generation of detrimental reactive oxygen species in Fenton and Haber–Weiss reactions [17]. Heme prosthetic groups present in many enzymes are more resistant to reactive oxygen species (ROS) than Fe-S clusters; heme-containing peroxidases that utilize H_2_O_2_ as obligatory co-substrates can serve as an example.

The intricate mechanisms of regulation of the intracellular iron levels that have been developed in mammalian cells evolved to maintain a fragile balance of the iron stores to satisfy the needs and limit the potential toxicity of ferrous ions. Cellular uptake of iron (via transferrin receptors or divalent metal transporter DMT1), its proper storage (in ferritin nano-cage), and export (through ferroportin) are tightly controlled. The important mechanism of regulation operates at the post-transcriptional level and engages iron regulatory proteins IRP1 and IRP2. Both iron regulatory proteins recognize and bind the stem-loop secondary structure motifs (so-called iron-responsive elements, IREs) in the mRNA molecules that encode for proteins involved in iron storage, transport, and metabolism (e.g., ferritin light and heavy subunits, transferrin receptor, ferroportin, 5-aminolevulinate synthase, succinate dehydrogenase, mitochondrial aconitase, etc.) [18]. The stem-loop structures are formed by the evolutionary conserved sequences present in either 5′- or 3′-UTR of the transcripts [19,20]. The general rule is that IRP binding to 5′-UTR IREs inhibits translation of the mRNA, whereas binding to 3′-UTR IREs protects the mRNA from degradation and sustains translation [21].

Labile iron pool (LIP) is defined as transitory, either Fe^2+^ or Fe^3+^ iron complexes capable of redox cycling, prone to scavenging by cell-permeable chelators [22]. LIP has the potential to elicit cytotoxicity by ROS generation, which eventually leads to the programmed cell death called ferroptosis. Ferroptosis has unique features that distinguish it from apoptosis, necrosis, and other known cell death modes [23]. The central process in ferroptosis is the peroxidation and oxidative damage of the membrane phospholipids.

Several circumstances highly increase the risk of ferroptosis, if they occur separately or in combination: (1) iron overload (e.g., after the treatment with iron ionophores) or a rapid release of iron from ferritin-protected pool during ferritinophagy (a lysosomal degradation of ferritin); (2) depletion of the intracellular glutathione stocks (insufficient glutathione synthesis); (3) inhibition of glutathione peroxidase GPX4, which is particularly effective in detoxifying the lipid peroxides embedded in phospholipid membranes; (4) increased incorporation of polyunsaturated fatty acyl chains into the membrane phospholipids [24].

The accumulation of lipid peroxides in the ER membranes is an early hallmark of ferroptosis, and the peroxidation further spreads to other organelle membranes (Golgi apparatus, mitochondria, lysosomes) and plasma membrane [25]. An intriguing feature of ferroptosis propagation in the cell population is a nonrandom, wave-like spreading pattern [26]. The propagation of lipid peroxidation and membrane damage among the cells takes place before the ultimate cell rupture and involves alterations in the intracellular calcium flux [26]. The cell death in this case results from the solute imbalance caused by the formation of several nanometer-sized pores in the plasma membrane [26], which therefore can be classified as a kind of regulated necrosis. The waves of ferroptosis are distributed both in the populations of cultured cells and tissues in a collective, organized fashion. Quick propagation of ferroptotic cell death over large distances (reaching 600 µm) within tissues shares characteristics with the so-called ‘bystander effect’ known from radiation therapy, where the death of irradiated cells spreads to the non-irradiated neighbors [27,28,29].

### 2.4. Copper

Like iron, copper ions participate in a variety of cellular processes acting as prosthetic groups or coenzymes for numerous enzymes, mostly oxidoreductases, due to their capability for redox cycling between cuprous (Cu^+^) and cupric (Cu^2+^) states. In mammalian cells, important examples of copper-dependent enzymes include oxidases involved in energy metabolism (e.g., cytochrome c oxidase), intermediate metabolism (diamine oxidases), melanin pigment formation (tyrosinase), neurotransmitter metabolism (dopamine β-hydroxylase), and ROS detoxification (Zn/Cu superoxide dismutase, SOD1). Due to a potential cytotoxicity associated with catalysis of the Fenton reaction, free copper ions are present intracellularly in extremely low, sub-femtomolar range of concentrations, whereas most copper ions (i.e., micromolar concentrations) are protein-bound [30]. This phenomenon also corroborates the order in the Irving–Williams series, where out of all biologically important divalent metal ions, copper–protein complexes are the most stable: Cu^2+^ > Zn^2+^/Ni^2+^ > Co^2+^ > Fe^2+^ > Mn^2+^ > Ca^2+^ > Mg^2+^ [31].

Dietary copper is absorbed by duodenal and small intestine epithelium by the apical plasma membrane transporter CTR1 and then released by ATP7A transporter ATPase in the basolateral membranes. In the blood, copper is bound to ceruloplasmin, albumin, and α2 macroglobulin and transcuprein [32,33,34]. After intracellular uptake, copper trafficking to organelles is mediated by cytosolic chaperones: CCS, which transfers Cu^+^ to cytosolic SOD1 and copper-dependent ATPase Atox1, mitochondrial copper chaperones (cytochrome oxidase assembly factors) Cox17, Cox19, and Cox23, metallothioneins, and ATP7A/B located in the trans-Golgi network [35,36,37].

A fraction of copper ions can also form a less tightly protein-bound population engaged in the regulation of protein activity. This phenomenon is called a metalloallostery, and its mode of action involves copper binding to non-catalytic sites (‘exosites’) [38]. Copper, being a transition redox reactive metal, similarly to iron, is inclined to evoke cytotoxicity; therefore, the labile copper pool is kept at extremely low concentrations [39]. Indeed, forced copper entry into cells by the application of copper ionophores such as elesclomol induces a newly discovered type of regulated cell death called cuproptosis [40]. Copper accumulation is particularly detrimental for the cells that actively respirate and rely on oxidative phosphorylation for energy purposes. Mitochondria are the main location of processes leading to the cuproptotic cell death [40].

### 2.5. Zinc

Zinc is an essential micronutrient, necessary for the structural maintenance and activity of numerous enzymes extremely important for individual cells, tissues, organs, and multicellular organism survival. The most prominent examples include enzymes engaged in metabolism (e.g., enolase, fructose-1,6-phosphate bis phosphatase, alcohol dehydrogenase, glycerol kinase), protein cleavage (matrix metalloproteinases, carboxypeptidase, leucine aminopeptidase, caspases), antioxidant protection (superoxide cytosolic dismutase SOD1, extracellular SOD3), circulation and tissue homeostasis (carbonic anhydrase, angiotensin-converting enzyme, alkaline phosphatase), as well as a broad and diverse group of regulatory proteins including zinc finger transcription factors (Snail, Slug, Kruppel-like factor, nuclear hormone receptors, sonic hegdehog, p53 tumor suppressor) [41,42,43]. Intracellular Zn^2+^ concentration range oscillates around 200–300 µM, whereas the free zinc pool is in the pico- or nanomolar range [44]. The import of zinc and its intracellular flux among various compartments are regulated by the membrane transporters ZnT (SLC30 family of proteins) and ZIP transporters. ZnT1 (SLC30A1) is localized in the plasma membrane, and the zinc transport from cytosol to Golgi is carried out by ZnT6 (SLC30A6), to lysosomes by ZnT2 and ZnT4 (SLC30A2 and SLC30A4) [45,46,47].

Apart from the role in catalysis, zinc ions participate in the intracellular signal transduction from the receptors, forming a so-called “zinc waves” of ions released from the ER [48]. The examples of cells that use zinc as a second messenger include mast cells and neurons, whereas in the brain, zinc can also act as a neurotransmitter [48,49,50].

Zinc accumulation in lysosomes is a part of the detoxification mechanism in case of zinc overload [51], but after exceeding the capacity of zinc storage, lysosomal membranes are prone to oxidative damage, which leads to lysosome membrane permeabilization (LMP), and release of hydrolases to the cytosol, leading to cell death. Cancer cells differ from healthy ones with respect to the lysosomal zinc balance: it was revealed that melanoma cells had very high levels of mucolipin TRP channel (TRPML1), which is a membrane ion channel for Ca^2+^ and Zn^2+^ transport [52]. TRPML1 facilitates zinc storage in lysosomes. Selective synthetic agonists of TRPML1 induce Zn^2+^ release to the cytoplasm, which results in a rapid loss of mitochondrial membrane potential and ATP depletion within 30–60 min. Necrotic cell death approaches in approximately 12 h after the agonist challenge. Importantly, this process, called ‘lysozincrosis’ by the authors, is completely absent in normal melanocytes [52].

Sulfur atoms in cysteine thiol groups function as ligand donors for zinc ions in metallothioneins (MTs), small (6 kDa) cysteine-rich proteins involved in essential metal storage and heavy metal sequestration and detoxification [53]. Apart from Zn^2+^, MTs can bind various metal ions, mostly Cu^+^ and Cd^2+^ [54,55]. Metallothioneins play diverse, not yet completely elucidated biological roles, such as universal metallochaperones (e.g., zinc suppliers) for various apoproteins (enzymes or zinc finger transcription factors), scavengers of Cu^+^ and other potentially toxic metal ions, essential metal ion reservoirs and storage places, and they also take part in the intracellular redox balance maintenance [56].

Other thiol-containing molecules, such as glutathione (GSH and GSSG), form complexes with zinc ions as well [57,58]. Notably, the intracellular GSH/GSSG ratio determines the rate and efficiency of zinc ion transfer from MTs to the zinc-dependent apoenzymes [59].

The expression of thionein (apoprotein lacking metals)-encoding genes is upregulated in mammals by various stress factors [60,61]. Accordingly, metal regulatory element (MRE) and glucocorticoid response element (GRE) sequences were identified in the thionein encoding genes [62,63].

## 3. The Basic Mechanisms Behind the Plastic Environmental Crisis

The scale and precise biological mechanisms of microplastic toxicity in humans are still largely unknown and remain the main subjects of many ongoing scientific projects. Particulate plastics are composed of theoretically biologically neutral polymers (polycarbonate, polystyrene, polyethylene, polyamide, and others) and various potentially toxic intentionally added chemicals. Many well-known functional additives (e.g., bisphenol A, parabens, phthalates, polybrominated flame retardants, heavy metals) improve some important features of manufactured materials by increasing their durability or resistance to physical and chemical degradation caused by light, temperature, or other environmental factors, or by altering products’ color and transparency [64]. In addition, due to chemical properties and uneven surface microstructure, particulate plastic has an enormous capacity to adsorb various organic and inorganic substances or metal ions present anyway in the environment. Therefore, very small nanoplastics are easily becoming effective vectors enabling the entrance of disruptive “passengers” into the interior of organisms or even particular tissues, cells, and organelles [65]. The superficial absorption of various molecules of environmental or organic origin is called ‘eco-’ or ‘bio-corona’ formation [66]. When the corona is formed by proteins, the suspension of plastic microparticles can be stabilized due to facilitated wettability, modified steric hindrance, and electrostatic repulsion [67]. Such a “protein corona” can mediate the bio accessibility of the metal ions [68,69].

MNPLs, due to their hydrophobic properties, interact with the cell surface and are internalized by cells through several mechanisms: passive penetration across the plasma membrane or active endocytosis that requires energy [70,71]. The experiments with polystyrene microbeads and rat basophilic leukemia RBL-2H3 cells revealed that the mechanisms of absorption depend on the size of particles: 50 nm particles undergo micropinocytosis, clathrin-mediated or caveolin-mediated endocytosis, whereas 500 nm particles are mainly internalized through micropinocytosis [70]. Large particles (approximately 5 μm) cannot adhere to the cell surface, which hampers their internalization [70]. Generally, in the case of the particles with a size exceeding 1 μm, phagocytosis is the main route of entry into cells [72]. Once the 50 nm and 500 nm particles enter the cells, they are distributed in the cytoplasm and targeted to lysosomes [70]. The lysosome-dependent active exocytosis is a major route of particle excretion, and the MNPL intracellular content decreases during the next 24–72 h [70,73]. This time period creates the possibility for the cells to respond with altered gene expression. The intracellular presence of MNPLs is likely to interfere with numerous processes. For instance, nanoplastic particles disturb protein folding and induce protein aggregation, which leads to proteotoxic stress [74]. All these actions of MNPLs have the potential to change cell behavior and physiology.

## 4. The Interaction of Metal Ions and Plastic Micro and Nanoparticles with the Human Gene Expression—Insight from the Available Databases and Online Tools

As discussed above, plastic particles, due to their hydrophobic nature, porosity, as well as chemical and physical modifications, serve as carriers of various molecules, including metals, into the tissues and organs of living organisms and individual cells. These features are especially enhanced in cases of particulate plastic weathering and ageing that have taken place [64,65]. Once internalized by cells, the plastic particles are likely to interact with proteins or phospholipids, but they may simultaneously release “stowaway passengers”, such as metal ions. Normally, phospholipid membranes are almost impermeable to metal ions, and their entrance via passive diffusion is a very slow process (diffusion coefficient for various ions is approximately 10^−18^ m^2^/s range [75]. In contrast, nanoplastic particles are internalized by cells within a time frame of seconds/minutes, probably as a result of association with phospholipid membranes and being taken along with ongoing endocytosis [76]. The mathematical model describing the leaching of metals present in MNPLs, applied to data from zebrafish exposed to Ag^+^-treated polyethylene microplastic beads, indicates that Ag^+^ attached to microplastic beads would be completely released in the fish organism within 1 h [77,78]. Once the metal-containing nanoparticles are taken up by cells, they are distributed to organelles and release metal ions with a diffusion coefficient range of 10^−15^–10^−20^ m^2^/s [70,77]; therefore, ions can reach multiple intracellular targets. In consequence, they may alter cell oxidative balance [79], mitochondrial function [80], and gene expression patterns [81].

### 4.1. The Retrieval of Genes Interacting with MNPLs and Metal Ions

#### 4.1.1. Methodology of the Search for Metal and MNPL Gene Targets

To gain a deeper insight into the potential outcomes of the gene expression alterations, we utilized the Comparative Toxigenomics Database (CTD, https://ctdbase.org/, accessed on 29 July 2025) [82]. The search tool ‘chemical-gene interaction query’ was applied to investigate the known interactions between the chemicals (metal ions, microplastics) and gene expression in humans. We used the chemical search terms “Iron”, “Copper”, “Zinc”, “Magnesium”, “Calcium”, and “Microplastics”, which refer to all the chemical polymer types, as the descendant compounds. The type of interactions was narrowed down to those that affect expression by selecting “expression” and all the boxes, including “increases”, “decreases”, and “affects (degree unspecified)”. The retrieved lists of genes that interact with the chemical specified as the search term [the lists are shown in Supplementary Figure S1, Supplementary File S1] were compared. We used the InteractiVenn online tool (https://www.interactivenn.net/, accessed on 29 July 2025) [83] to filter for the common genes. The resulting sets of common genes were analyzed with the Metascape platform (https://metascape.org/gp/index.html#/main/step1, accessed on 29 July 2025) [84] and the STRING database (https://string-db.org/, accessed on 29 July 2025) [85], which perform gene ontology annotations and group genes in functional clusters.

#### 4.1.2. The Analysis of the Retrieved Genes

**Figure 1:**
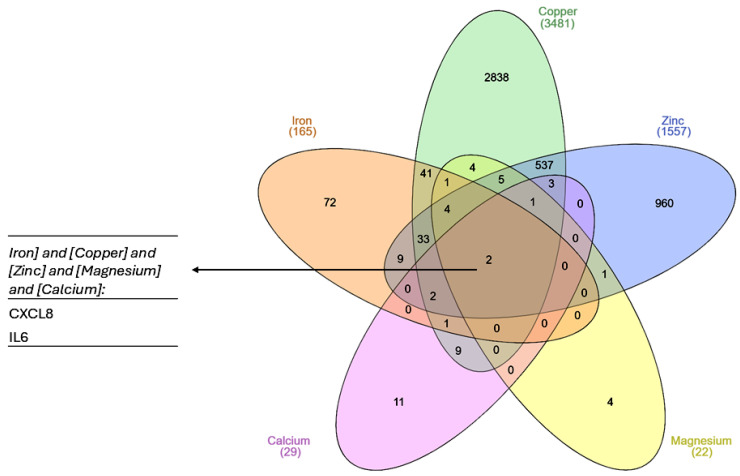
A Venn diagram showing common genes for each of the list of metal-interacting genes retrieved from the CTD. The plot was created with InteractiVenn online tool [83].

Only two genes were found to interact with all the metals (Figure 1): IL6 and CXCL8, encoding interleukin 6 and C-X-C motif chemokine ligand 8 (monocyte-derived neutrophil chemotactic factor). Both genes are involved in the acute phase of inflammation and the innate immune response, suggesting that a common feature of exposure to metal ions is the augmentation of inflammatory processes.

### 4.2. Assigning the Biological Meaning to the Revealed Set of Genes

**Figure 2:**
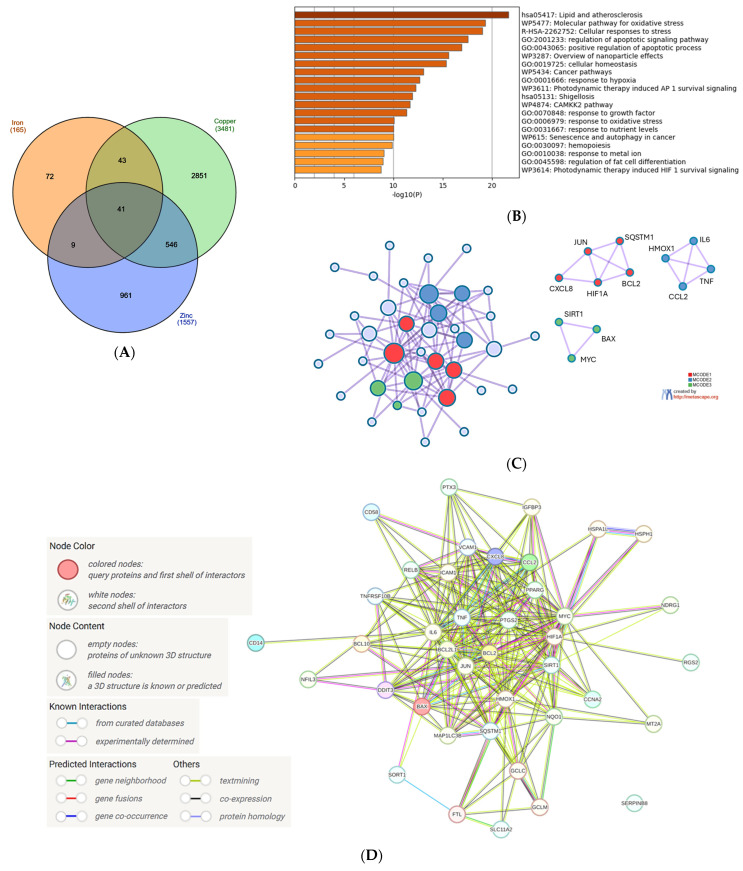

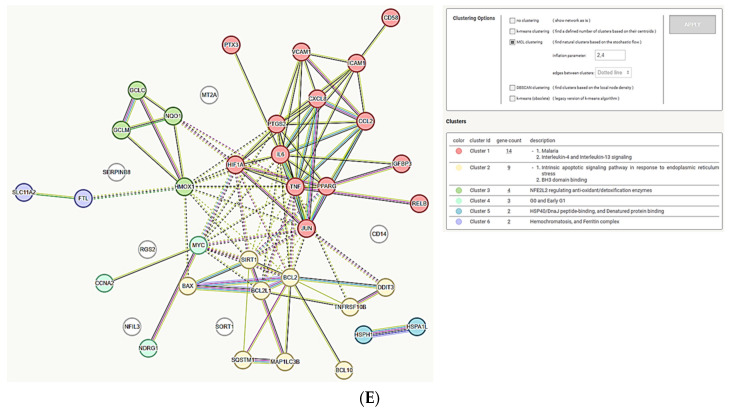
Analysis of the common genes regulated by iron, copper, and zinc. (**A**) Venn diagram of the gene sets retrieved in the CTD by searching the gene interactions with iron, copper, and zinc. The list of the 41 genes is in Supplementary File S2. The plot created with InteractiVenn online tool [83]; (**B**) analysis of the 41 gene set with Metascape platform [84]. The list of 20 top-level gene ontology processes with the lowest *p* values. The colors indicate the range of *p* values: for log10(*p*) < −20 dark brown color is used, for −20 < log10(*p*) −10 light brown is used, for log10(*p*) > −10 orange is used; (**C**) gene clusters retrieved from Metascape platform. MCODE1 (red cluster) annotated to “Senescence and autophagy in cancer” [log10(*p*) = −9.2], “Signaling by interleukins” [log10(*p*) = −9.1], “Cytokine signaling in immune system” [log10(*p*) = −8.0]; MCODE2 (blue cluster) annotated to “Antiviral and anti-inflammatory effects of Nrf2 on SARS-CoV-2 pathway” [log10(*p*) = −11.9], “Lung fibrosis” [log10(*p*) = −10.8], “COVID adverse outcome pathway” [log10(*p*) = −9.3]; MCODE3 (green cluster) annotated to “Positive regulation of leukocyte apoptotic process” [log10(*p*) = −8.9], “Positive regulation of intrinsic apoptotic signaling pathway: [log10(*p*) = −8.0], PID p73 pathway”(p73 transcription factor network) [log10(*p*) = −7.8]; (**D**) analysis of the interactions within the set of 41 genes in STRING database [85]. Network nodes represent proteins produced by a single protein-encoding gene locus. Edges represent protein–protein interactions that are meant to be specific and meaningful, i.e., proteins jointly contribute to a shared function, but they do not necessarily bind physically; (**E**) gene clustering analysis (Markov Cluster Algorithm method, MCL) in the STRING database.

The small number of genes common to all metals made the investigation of the interference with the set of microplastic-modulated genes irrelevant. Therefore, it prompted us to focus on iron, copper, and zinc, which exhibit a high and diverse biological activity and whose intracellular levels are tightly regulated due to possible toxicity. The analysis revealed 41 genes with the expression regulated by and common to all three metals (Figure 2A). This set of genes was next uploaded into the Metascape platform.

The first 5 biological processes with the lowest false discovery rate (FDR) measure [−log10(*p*) > 15] included ‘Lipid and atherosclerosis’, ‘Molecular pathway for oxidative stress’, ‘Cellular response to stress’, ‘Regulation of apoptotic signaling pathway’ and ‘Positive regulation of apoptotic process’ (Figure 2B). These processes, especially connected to response to stress and apoptosis, seem to fit well into the current knowledge about the impact of iron, copper and zinc on cellular physiology. Interestingly, the sixth position with a very high FDR is ‘Overview of nanoparticle effects’, which encouraged us to further analyze the interactions between the genes modulated by metals and MNPLs (see Section 4.3).

The clusters revealed by Metascape comprised three main pathways: (1) the pathway involved in cytokine/interleukin signaling in immune system; senescence and autophagy in cancer; (2) antiviral and ani-inflammatory signaling of Nrf2 in SARS-CoV-2, lung fibrosis and COVID-19-adverse outcome pathway; and (3) positive regulation of apoptotic processes in leukocytes and intrinsic apoptotic pathways (Figure 2C). The Metascape analysis was supported by the gene interconnection analysis in STRING database [85]: the core of the network is formed by the genes strongly engaged in the inflammatory processes of the immune system (IL6, TNF, PTGS2, JUN) and the regulation of apoptosis (BCL2, BCL2L1, Figure 2D). The cluster analysis performed in the STRING database grouped the genes into six clusters, with the two biggest ones involved in interleukin 4 and interleukin 13 signaling and the intrinsic apoptotic response to ER stress (Figure 2E). Not surprisingly, this analysis also revealed a cluster involved in iron metabolism: hemochromatosis and ferritin complex (Figure 2E). Metallothionein 2A (MT2A) gene, encoding an evolutionary conserved protein synthesized in response to metal exposure [86], was present in the network, but not classified into any cluster (Figure 2E).

### 4.3. Analysis of the Gene Sets Modulated by Metals and MNPLs

**Figure 3:**
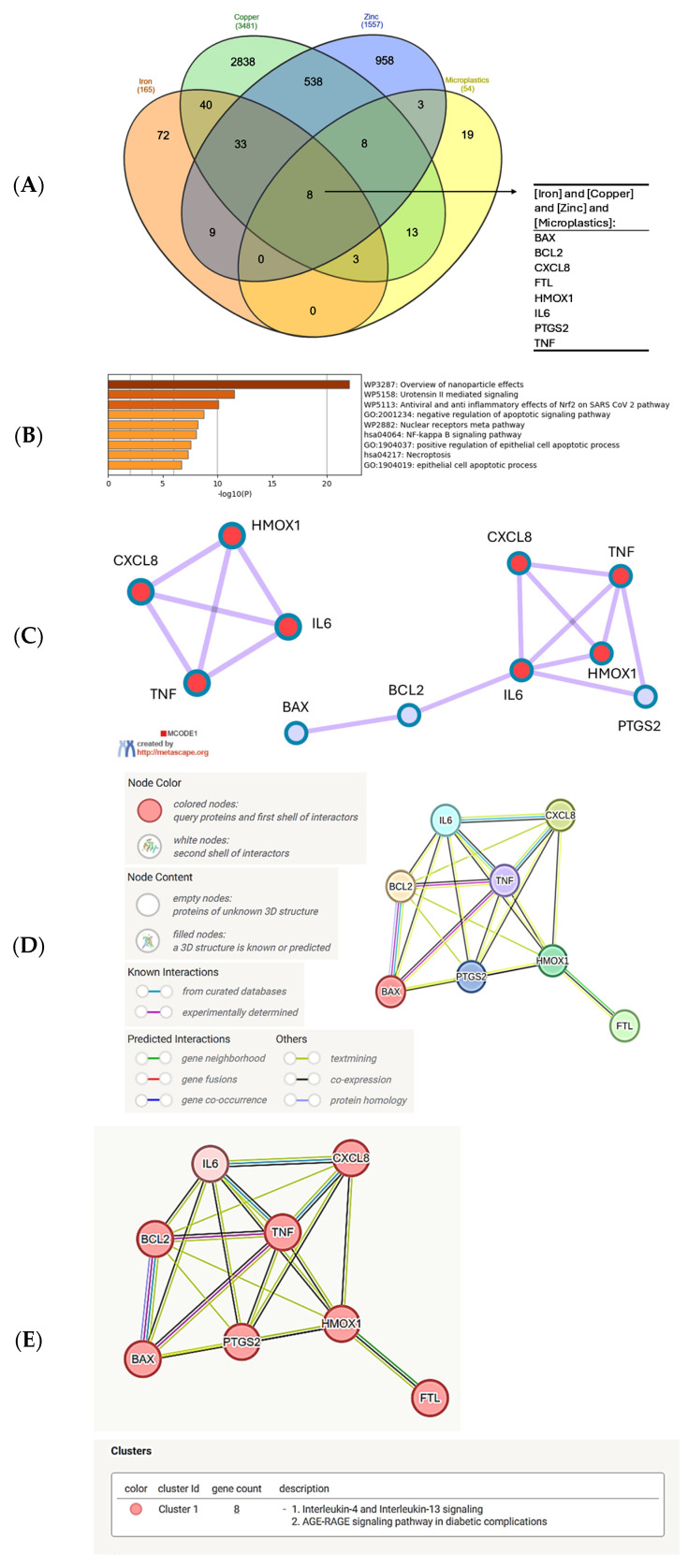
Analysis of the common genes regulated by iron, copper, zinc, and MNPLs. (**A**) Venn diagram of the gene sets retrieved in the CTD by searching the gene interactions with “Iron”, “Copper”, “Zinc”, and “Microplastics”. The list of the sets of genes common to microplastics and the metals is shown in the Supplementary File S2. The plot created with the InteractiVenn online tool [83]; (**B**) analysis of the 8 gene set with the Metascape platform [84]. The list of top-level gene ontology processes with the lowest *p* values. The colors indicate the range of *p* values: for log10(*p*) < −20 dark brown color is used, for −20 < log10(*p*) −10 light brown is used, for log10(*p*) > −10 orange is used; (**C**) gene cluster retrieved from the Metascape platform [84]. MCODE1 annotated to “Overview of nanoparticle effects” [log10(*p*) = −13.0], “Antiviral and anti-inflammatory effects of Nrf2 on SARS-CoV-2 pathway” [log10(*p*) = −11.9], “Molecular pathway for oxidative stress” [log10(*p*) = −11.2]; (**D**) analysis of the interactions within the set of 8 genes in STRING database [85]. Network nodes represent proteins produced by a single protein encoding gene locus. Edges represent protein–protein interactions that are meant to be specific and meaningful, i.e., proteins jointly contribute to a shared function, but they do not necessarily bind physically; (**E**) gene clustering analysis (Markov Cluster Algorithm method, MCL, STRING database). All the genes belong to one cluster.

Next, we confronted the set of genes common to the response to iron, copper, and zinc with the set of genes interacting with MNPLs. We obtained the group of 8 genes that were previously found in the center or proximity of the center in the metal gene network, namely: BAX, BCL2, CXCL8, FTL, HMOX1, IL6, PTGS2 and TNF (in alphabetical order, Figure 3A). As was expected, the Metascape analysis of this set of genes indicated the top-score biological process as ‘Overview of nanoparticle effects’ (Figure 3B). The next two processes with much lower scores include ‘Urotensin II mediated signaling’ and ‘Antiviral and anti-inflammatory signaling of Nrf2 in SARS-CoV-2 pathway’. Urotensin II is a cyclic peptide of neurohormonal activity, very highly conserved among vertebrates [87]. It is a very potent vasoconstrictor, signaling through the G-protein-coupled GPR14 receptor, which is important for the function of the cardiovascular system and kidneys. It also mediates the synthesis of proinflammatory cytokines TNFα, IL-1β, IL-8, and lipid mediators, such as leukotriene C4 [88,89]. Therefore, the presence of numerous cytokine genes in the network analyzed here strongly justifies the annotation to urotensin II signaling and the COVID-19-associated pathway.

Inflammation is a local or systemic response to stress and tissue injury. Inflammatory action of numerous immune cell populations, such as monocytes, macrophages, and granulocytes, involves the release of reactive oxygen and nitrogen species (ROS, RNS). The cellular protection from oxidative stress is mediated by nuclear factor erythroid 2-related factor 2 (Nrf2), which is a transcription factor activating the expression of ROS/RNS detoxifying genes, including glutathione S-transferase, NAD(P)H: quinone oxidoreductase 1 (NQO1), thioredoxin reductase 1 (Txnrd1), and heme oxygenase (HO-1) [90]. The connection to the Nrf2 pathway is rationalized by the presence of the heme oxygenase 1 gene (HMOX1) in the network of the metal–microplastic interacting genes (Figure 3C–E). Therefore, oxidative stress evoked by the innate immune mechanisms, the intrinsic protective mechanisms, and inflammation are all connected in the gene set common to iron, copper, zinc, and microplastics (Figure 3E).

**Figure 4:**
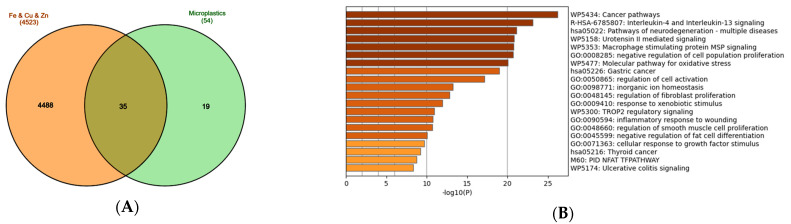

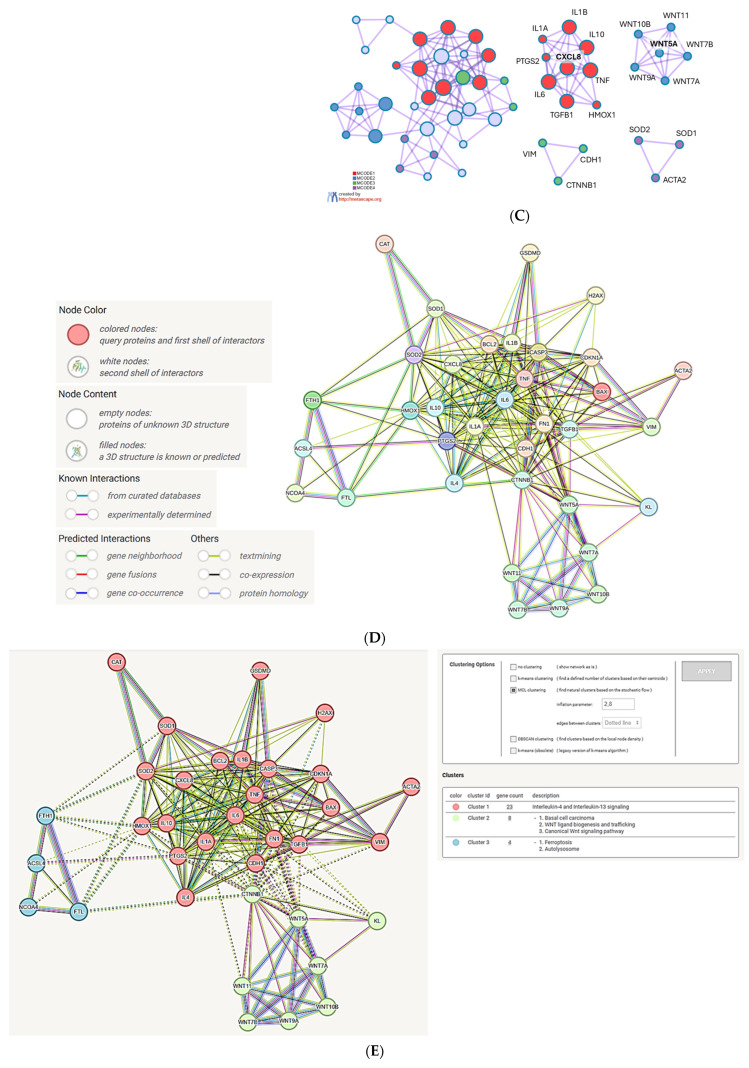
Analysis of the genes regulated by metals (iron, copper, zinc) and common to MNPLs. (**A**) Venn diagram of the gene sets retrieved in the CTD [82] by searching the gene interactions with “Iron”, “Copper”, “Zinc”, and “Microplastics”. The sum of genes that interact with iron, copper, and zinc (4523 genes in total) was compared to the 54-gene set that interacts with microplastics. The set of 35 common genes (the list in the Supplementary File S2) was retrieved. The plot created with the InteractiVenn online tool [83]; (**B**) analysis of the 35 gene set with the Metascape platform [84]. The list of the 20 top-level gene ontology processes with the lowest *p* values. The colors indicate the range of *p* values: for log10(*p*) < −20 dark brown color is used, for −20 < log10(*p*) −10 light brown is used, for log10(*p*) > −10 orange is used; (**C**) gene clusters retrieved from the Metascape platform. MCODE1 (red cluster) annotated to “IL-4 and IL-13 signaling” [log10(*p*) = −18.8], “Post COVID neuroinflammation” [log10(*p*) = −17.0], “Cytokines and inflammatory response” [log10(*p*) = −16.7]; MCODE2 (blue cluster) annotated to “WNT ligands biogenesis and trafficking” [log10(*p*) = −18.7], “Basal cel carcinoma” [log10(*p*) = −16.2], “Polycystic kidney disease” [log10(*p*) = −15.4]; MCODE3 (green cluster) annotated to “TROP2 regulatory signaling” [log10(*p*) = −8.5], “Macrophage stimulatory protein signaling” [log10(*p*) = −7.4], “Cellular response to nitrogen compounds” [log10(*p*) = −5.1]; MCODE4 (Purple cluster) annotated to “Blood vessel diameter” [log10(*p*) = −6.8], “Regulation of tube diameter” [log10(*p*) = −6.8], “Regulation of tube size” [log10(*p*) = −6.8]; (**D**) analysis of the interactions within the set of 35 genes in STRING database [85]. Network nodes represent proteins produced by a single protein encoding gene locus. Edges represent protein–protein interactions that are meant to be specific and meaningful, i.e., proteins jointly contribute to a shared function, but they do not necessarily bind physically; (**E**) gene clustering analysis (Markov Cluster Algorithm method, MCL, STRING database).

The environmental exposure to all three metals, Fe, Cu, and Zn, in the presence of plastic particle carriers, does not necessarily mean that all these metals are present at the same time. Therefore, we decided to check out the broader interactions by identifying the set of genes common to the sum of all genes interacting with the individual metals and microplastic-interacting genes. The Venn diagram (Figure 4A, Supplementary File S2) shows 35 such genes, which were next subjected to the Metascape analysis. Seven biological processes with the highest FDR score (with values exceeding 20) indicate the previously revealed inflammation and the activity of the innate immune system (urotensin II signaling) and the oxidative stress pathways (Figure 4B). This time, the immune system reaction, apart from the pro-inflammatory urotensin II signaling, is focused on alternative polarization of macrophages (IL-4 and IL-13 signaling) and pro-regenerative mechanisms that engage macrophage stimulatory protein (MSP) [91,92]. The commitment to tissue remodeling, regeneration, and wound healing seems comforting and harmless, although it might also indicate engagement in fibrosis and scar tissue formation [93]. Fibrotic scar tissue may not fully perform physiological functions, and in some cases, may have negative consequences.

In certain conditions, such as neoplastic transformation, alternatively activated macrophages are not favorable, since they interfere with the proper cytotoxic response and elimination of the transformed cells by the immune system. Tumor-associated M2 polarized macrophages contribute to the cancer progression through promoting angiogenesis and blunting anti-tumor immunity of the CD8+ T cells and NK-cells [94]. Indeed, the gene ontology analysis revealed the alarming involvement in pathological processes such as cancer (the most enriched ‘Cancer pathways’ annotation).

Inflammatory processes within the central nervous system that involve activation of microglia and astrocytes are termed neuroinflammation. They commonly contribute to the development of neurodegenerative diseases [95]. The inflammatory genes present in the network reflect in the high position of ‘Pathways of neurodegeneration’ (Figure 4B) and the cluster ‘Post COVID-19 neuroinflammation’ (Figure 4C).

The other clusters include a group of Wnt-encoding genes, WNT5A, WNT7A, WNT7B, WNT9A, WNT10B, WNT11, which are important for embryonic development, determine cell fate, and are required for the maintenance of cell renewal and regenerative potential in adult organisms [96,97]. The dysregulation of their function may lead to neoplastic transformation and cancer development, particularly basal and squamous cell carcinoma of the skin [98,99]. The additional two clusters in Metascape analysis include previously mentioned ‘Macrophage stimulatory protein signaling’ and ‘Blood vessel diameter’, which most probably are connected to urotensin II vasoconstricting activity (Figure 4C).

The network obtained with the STRING database tool (Figure 4D) shows the highest number of connections between the inflammatory genes (TNF, IL6, IL1B, CXCL8), the genes involved in the connective tissue development, regeneration, and remodeling (FN1, CDH1, TGFB1, CTNNB1), and the apoptosis-related genes (BAX, BCL2, CASP3). On the periphery of the network, there are clearly defined groups of genes involved in antioxidant protection (HMOX1, SOD1, SOD2, CAT), ferroptosis (ACSL4, NCOA4, FTL, FTH1) and WNT protein family (Figure 4D). The cluster analysis confirmed the affiliation of the genes to three clusters defined before: (1) ‘IL-4 and IL-13 signaling’; (2) ‘Basal cell carcinoma, WNT ligand biogenesis, canonical WNT signaling’ and (3) ‘ferroptosis and autolysosome’ (Figure 4E).

The latter cluster supports the notion that MNPLs, by facilitating metal ion uptake by cells, might contribute to certain metal-induced cell death types. The genes from the ferroptosis cluster comprise Acyl-CoA Synthetase Long Chain Family Member 4 (ACSL4), Nuclear Receptor Coactivator 4 (NCOA4), as well as ferritin light and heavy chains (FTL, FTH1). ACSL4 participates in the synthesis of membrane phospholipids enriched in polyunsaturated fatty acyl chains, particularly arachidonic acid, which is prone to peroxidation due to the presence of double bonds [100]. NCOA4 is a cargo receptor responsible for ferritinophagy, a process of the selective degradation of ferritin nanocage formed by FTL and FTH, through its delivery into lysosomes. The transfer to lysosomes leads to ferritin degradation and release of iron stores [101]. NCOA4 directly binds to ferritin and colocalizes with ferritin in autophagosomes and lysosomes [102]. The NCOA4-deficient cells show much higher resistance to ferroptosis than wild-type cells [103]. Similarly, the genetic knock out of ACSL4 protects from ferroptosis [104].

The existence of the ferroptosis cluster supports the idea that MNPLs might serve as carriers that facilitate the entry of iron into the cells, which have difficulty coping with oxidative stress. Despite the launching of oxidative stress-responsive genes, such as HMOX1, SOD, and CAT, the cells are prone to oxidative damage by transition metal-induced ROS.

## 5. Discussion

So far, there is no strong evidence that MNPLs are carcinogenic per se, in the sense that they act as initiators of cancer development by genotoxic or mutagenic activity. Nevertheless, due to the role of carriers of pollutants of various origins, such as carcinogenic compounds, polycyclic aromatic hydrocarbons (PAHs), persistent organic pollutants (POPs), heavy metals, hormone-mimicking substances such as bisphenol A (BPA), resembling estrogens, these might contribute to the neoplastic transformation [105,106,107]. MNPLs can promote the molecular events that accelerate cancer progression by secondary mechanisms, such as interference with signal transduction pathways and promoting inflammatory response [108,109].

By affecting gene expression patterns, particulate plastics may impair the functioning of organs and systems. A recent study demonstrated that a long-term (12 weeks) exposure to NMPLs induces liver fibrosis in mice, with increased expression of the α-smooth muscle actin (ACTA2) gene [110]. The authors identified three hub genes: acyl-CoA thioesterase 3 (ACOT3), ATP-binding cassette subfamily C member 3 (ABCC3), also known as MRP3, and Nuclear receptor subfamily 1 Group I member 3 (NR1I3) [110]. These genes coordinate lipid metabolism and are candidate molecular targets of NMPLs in the liver [110]. Our analysis also revealed the connections to the genes associated with fibrotic remodeling, namely TGFB1, ACTA2, and VIM (Figure 4E), among the genes that interact with metals and microplastics.

Interestingly, the ABCC3 gene, identified as the hub in the study by Li et al. [110], encodes for the protein transporter that exports various lipid-related and hydrophobic compounds outside the cell. ABCC3 is involved in multidrug resistance and serves as a chemotherapeutic drug resistance tool (vincristine, etoposide, cisplatin, doxorubicin, methotrexate) in non-small lung cancer [111,112,113,114].

MNPLs have also been shown to increase multidrug resistance to bortezomib, paclitaxel, gefitinib, lapatinib, and trastuzumab in gastric cancer cell lines and gastric cancer murine model in vivo [115]. The molecular mechanism behind this effect involved the increased expression of CD44, a cancer stem cell marker in numerous human solid tumors [116], and subsequent upregulation of asialoglycoprotein receptor 2 (ASGRA2), which acts as an oncogene in gastric cancer [115]. In summary, all these recent works point to the mechanisms that underlie the involvement of microplastic exposure and carcinogenesis.

Solid microplastic beads have not been used as clinically approved drug carriers in cancer therapies so far. Particles made of various polymers (mainly the polyvinyl alcohol-based or tris-acryl gelatin microspheres) have been introduced and approved for clinical use during the last two decades, mainly as embolization agents used to block a tumor’s blood vessel supply (e.g., Bead Block^®^ of Boston Scientific Corporation or Embosphere^®^ of Merit Medical Systems). However, such particles cannot be classified as microplastics in the sense of environmental pollutants. The embolization agents are made of elastic, deformable, hydrophilic, and preferably easily biodegradable materials [117]. In addition, some metal-containing nanoparticles have been applied to anti-cancer treatment, taking advantage of their passive targeting into the tumor due to the imperfect structure of its vasculature and poor lymphatic drainage [118]. NanoTherm^®^ particle-containing amino silane-coated iron oxide (magnetite) nanoparticles were approved by the European Medicines Agency (EMA) and the US Food and Drug Administration (FDA) for the treatment of aggressive malignancies, such as glioblastoma and prostate cancer [118,119]. The purpose is the induction of hyperthermia inside tumor tissue by the action of an alternating magnetic field (AMF) on iron oxide nanoparticles. AMF is applied in the specified region, which leads to tumor cell death (thermoablation) [120]. Even in the absence of the heat effect, iron oxide nanoparticle preparation ferumoxytol has been shown to inhibit the growth of primary and metastatic mammary adenocarcinoma in mice by launching pro-inflammatory M1/Th1 polarized immune response [121].

Copper-based nanoparticles and copper-containing molecules are also regarded as promising therapeutic modalities against breast cancer and numerous other malignancies [122,123]. These novel chemical agents are designed to have diverse modes of action, such as induction of cytotoxic oxidative stress, various types of cell death, or targeting tumor angiogenesis [123]. An example of such an approach is chemo dynamic therapy (CDT) in drug-resistant breast cancer cells, using self-assembled copper-amino acid mercaptide nanoparticles (Cu-Cys NPs) [124]. After endocytosis into tumor cells, these nanoparticles react with GSH and deplete their local stores, while being reduced to the cuprous (Cu^+^) state. Cuprous ions trigger the Fenton reaction in situ and generate free radicals that kill tumor cells [124].

## 6. Conclusions

In summary, the interplay between MNPL-induced and metal-activated cellular responses is complex and affects numerous mechanisms, mainly antioxidant response, inflammatory processes, and tissue remodeling pathways. The large body of literature suggests that metal-associated MNPLs acting as metal ion carriers exert even more pleiotropic effects on healthy and transformed cells. Some of these effects need to be regarded as health hazards, but others might open new treatment opportunities against neoplastic diseases.

## Data Availability

All the data are included in the Supplementary Materials.

## Supplementary Materials

The following supporting information can be downloaded at https://www.mdpi.com/article/10.3390/biom15101418/s1: Figure S1: The chemical–gene interactions retrieved from the Comparative Toxigenomic Database (CTD) [access date: 29 July 2025]; File S1: The lists of genes retrieved from CTD for iron, copper, zinc, calcium, magnesium and microplastics; File S2: The lists of the common genes for Figure 2A, Figure 3A and Figure 4A.

## Abbreviations

The following abbreviations are used in this manuscript:

ABCC3	ATP-binding Cassette Subfamily Member 3
ACO	Aconitase
ACOT3	Acyl-CoA Thioesterase 3
ACSL4	Acyl-CoA Synthetase Long Chain Family Member 4
AMF	Alternating Magnetic Field
ASGRA2	Asialoglycoprotein Receptor 2
BCKDH	Branched Chain Ketoacid Dehydrogenase
BPA	Bisphenol A
CCS	Copper Chaperone for Superoxide Dismutase
CDT	Chemo Dynamic Therapy
CDT	Comparative Toxigenomic Database
DAG	Diacylglycerol
DMT1	Divalent Metal Transporter 1
EMA	European Medicines Agency
ER	Endoplasmic reticulum
Erk1,2	Extracellular Signal-Regulated kinase 1, 2
FDA	Food and Drug Administration
FDR	False Discovery Rate
FDX	Ferredoxin
FTH	Ferritin Heavy Chain
FTL	Ferritin Light Chain
GCSH	Glycine Cleavage System
GPX4	Glutathione Peroxidase 4
GRE	Glucocorticoid Responsive Element
GSH	Glutathione (reduced)
GSSG	Glutathione (oxidized)
HO-1	Heme Oxygenase 1
Hr	Hemerythrin-like domain
IL	Interleukin
IP3	Inositol 1,4,5-triphosphate
IRE	Iron Responsive Element
IRP1, 2	Iron Regulatory Protein 1, 2
LIAS	Lipoic Acid Synthase
LIP	Labile Iron Pool
LMP	Lysosomal Membrane Permeabilization
LPT1	Lipoyl Transferase 1
MCU	Mitochondrial Calcium Uniporter
MEK	Mitogen-Activated Extracellular Signal-Regulated Kinase
MNPLs	Micro- and nanoplastics
mPTP	Mitochondrial Permeability Transition Pore
MRE	Metal Regulatory Element
MSP	Macrophage Stimulating Protein
MT	Metallothionein
NCOA4	Nuclear Receptor Coactivator 4
NOX	NADPH Oxidase
NQO1	NAD(P)H: quinone oxidase 1
Nrf2	Nuclear Factor Erythroid 2–Related Factor 2
PAHs	Polycyclic Aromatic Hydrocarbons
PDH	Pyruvate Dehydrogenase
PI3K	Phosphoinositide 3-kinase
PKC	Protein Kinase C
PLC	Phospholipase C
POPs	Persistent Organic Pollutants
RNS	Reacive Nitrogen Species
ROS	Reactive Oxygen Species
SOD	Superoxide Dismutase
TNFα	Tumor Necrosis Factor α
TRPML1	Transient Receptor Potential Channel Mucolipin 1
ULK	Unc-51-Like Kinase
UTR	Untranslated Region
WHA	World Health Assembly
WNT	Wingless-related Integration Site
ZIP	Zrt-/Irt-Like Protein
