# Supplementary material for: Micro- and Nanoplastics Act as Metal Carriers with the Potential to Alter Human Gene Expression Patterns—The Inferences from Bioinformatic Online Tools
Source: Biomolecules. 2025 Oct 6;15(10):1418. doi: 10.3390/biom15101418 (PMC12564244; doi:10.3390/biom15101418)
Supplement: Supplementary file 1 [file biomolecules-15-01418-s001.zip › Biomolecules_Suppl_Material_Fig S1.pdf]

Supplementary materials

Supplementary Figure S1. The chemical – gene interactions retrieved from the Comparative Toxigenomic Database [ref, access date: 29.07.2025, screen shots] using the search term "*Iron*", "*Copper*", "*Zinc*", "*Magnesium*", "*Calcium*" and "*Microplastics*" (the search term with the descendants).

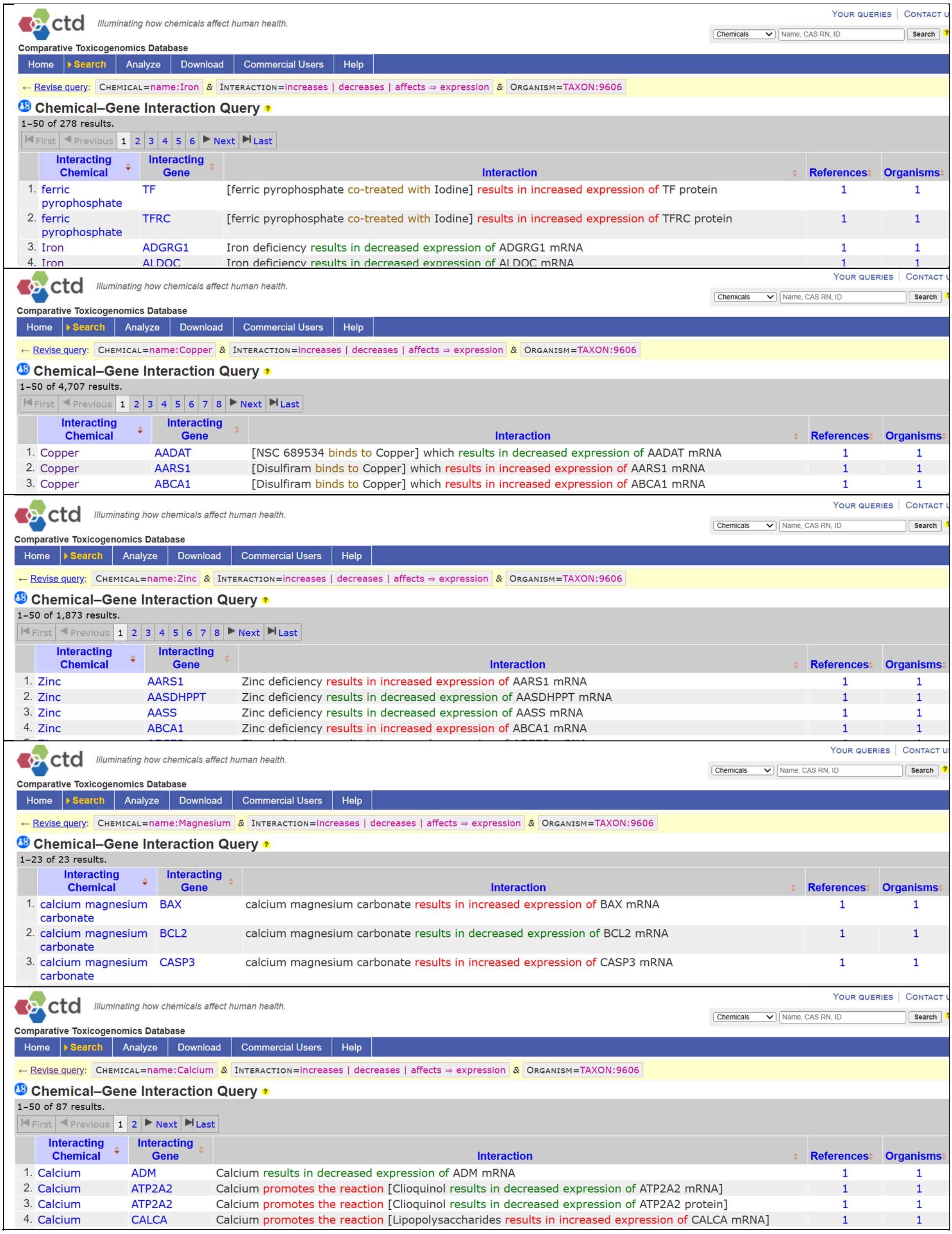

ctd Illuminating how chemicals affect human health.
Comparative Toxicogenomics Database

Home | Search | Analyze | Download | Commercial Users | Help

← Revise query: CHEMICAL=name:Iron & INTERACTION=increases | decreases | affects ⇒ expression & ORGANISM=TAXON:9606

Chemical–Gene Interaction Query

1–50 of 278 results.

First | Previous | 1 | 2 | 3 | 4 | 5 | 6 | Next | Last

	Interacting Chemical	Interacting Gene	Interaction	References	Organisms
1.	ferric pyrophosphate	TF	[ferric pyrophosphate co-treated with Iodine] results in increased expression of TF protein	1	1
2.	ferric pyrophosphate	TFRC	[ferric pyrophosphate co-treated with Iodine] results in increased expression of TFRC protein	1	1
3.	Iron	ADGRG1	Iron deficiency results in decreased expression of ADGRG1 mRNA	1	1
4.	Iron	ALDOC	Iron deficiency results in decreased expression of ALDOC mRNA	1	1

ctd Illuminating how chemicals affect human health.
Comparative Toxicogenomics Database

Home | Search | Analyze | Download | Commercial Users | Help

← Revise query: CHEMICAL=name:Copper & INTERACTION=increases | decreases | affects ⇒ expression & ORGANISM=TAXON:9606

Chemical–Gene Interaction Query

1–50 of 4,707 results.

First | Previous | 1 | 2 | 3 | 4 | 5 | 6 | 7 | 8 | Next | Last

	Interacting Chemical	Interacting Gene	Interaction	References	Organisms
1.	Copper	AADAT	[NSC 689534 binds to Copper] which results in decreased expression of AADAT mRNA	1	1
2.	Copper	AARS1	[Disulfiram binds to Copper] which results in increased expression of AARS1 mRNA	1	1
3.	Copper	ABCA1	[Disulfiram binds to Copper] which results in increased expression of ABCA1 mRNA	1	1

ctd Illuminating how chemicals affect human health.
Comparative Toxicogenomics Database

Home | Search | Analyze | Download | Commercial Users | Help

← Revise query: CHEMICAL=name:Zinc & INTERACTION=increases | decreases | affects ⇒ expression & ORGANISM=TAXON:9606

Chemical–Gene Interaction Query

1–50 of 1,873 results.

First | Previous | 1 | 2 | 3 | 4 | 5 | 6 | 7 | 8 | Next | Last

	Interacting Chemical	Interacting Gene	Interaction	References	Organisms
1.	Zinc	AARS1	Zinc deficiency results in increased expression of AARS1 mRNA	1	1
2.	Zinc	AASDHPPT	Zinc deficiency results in decreased expression of AASDHPPT mRNA	1	1
3.	Zinc	AASS	Zinc deficiency results in decreased expression of AASS mRNA	1	1
4.	Zinc	ABCA1	Zinc deficiency results in increased expression of ABCA1 mRNA	1	1

ctd Illuminating how chemicals affect human health.
Comparative Toxicogenomics Database

Home | Search | Analyze | Download | Commercial Users | Help

← Revise query: CHEMICAL=name:Magnesium & INTERACTION=increases | decreases | affects ⇒ expression & ORGANISM=TAXON:9606

Chemical–Gene Interaction Query

1–23 of 23 results.

	Interacting Chemical	Interacting Gene	Interaction	References	Organisms
1.	calcium magnesium carbonate	BAX	calcium magnesium carbonate results in increased expression of BAX mRNA	1	1
2.	calcium magnesium carbonate	BCL2	calcium magnesium carbonate results in decreased expression of BCL2 mRNA	1	1
3.	calcium magnesium carbonate	CASP3	calcium magnesium carbonate results in increased expression of CASP3 mRNA	1	1

ctd Illuminating how chemicals affect human health.
Comparative Toxicogenomics Database

Home | Search | Analyze | Download | Commercial Users | Help

← Revise query: CHEMICAL=name:Calcium & INTERACTION=increases | decreases | affects ⇒ expression & ORGANISM=TAXON:9606

Chemical–Gene Interaction Query

1–50 of 87 results.

First | Previous | 1 | 2 | Next | Last

	Interacting Chemical	Interacting Gene	Interaction	References	Organisms
1.	Calcium	ADM	Calcium results in decreased expression of ADM mRNA	1	1
2.	Calcium	ATP2A2	Calcium promotes the reaction [Clioquinol results in decreased expression of ATP2A2 mRNA]	1	1
3.	Calcium	ATP2A2	Calcium promotes the reaction [Clioquinol results in decreased expression of ATP2A2 protein]	1	1
4.	Calcium	CALCA	Calcium promotes the reaction [Lipopolysaccharides results in increased expression of CALCA mRNA]	1	1

ctd *Illuminating how chemicals affect human health.*

Comparative Toxicogenomics Database

YOUR QUERIES | CONTACT

Chemicals | Name, CAS RN, ID | Search

Home | Search | Analyze | Download | Commercial Users | Help

← Revise query: CHEMICAL=name:Microplastics & INTERACTION=increases | decreases | affects ⇒ expression & ORGANISM=TAXON:9606

Chemical–Gene Interaction Query

1–50 of 119 results.

First | Previous | 1 | 2 | 3 | Next | Last

	Interacting Chemical	Interacting Gene	Interaction	References	Organisms
1.	Microplastics	ACSL4	ferrostatin-1 inhibits the reaction [[Polystyrenes results in increased abundance of Microplastics] which results in increased expression of ACSL4 mRNA]	1	1
